# Substance‐Gain Extrusion Technique (SGET) in Combination With BOPT—Technique Description

**DOI:** 10.1111/jerd.13494

**Published:** 2025-05-19

**Authors:** M. Pirc, D.S. Thoma, L. Mancini, R.E. Jung, F.J. Strauss

**Affiliations:** ^1^ Clinic of Reconstructive Dentistry Center for Dental Medicine, University of Zurich Zurich Switzerland; ^2^ Universidad Autonoma de Chile Santiago Chile

**Keywords:** biologically oriented preparation technique (BOPT), dental implants, restorative dentistry, surgical extrusion, tooth extrusion

## Abstract

**Objective:**

The restoration of teeth with deep subgingival fractures poses a significant challenge, often requiring extensive interventions such as orthodontic extrusion or implant therapy. This manuscript aims to introduce and describe the substance‐gain extrusion technique (SGET) as a minimally invasive alternative that enables the preservation of natural dentition while optimizing biological and esthetic outcomes. By combining SGET with the biologically oriented preparation technique (BOPT), this approach seeks to address both functional and soft tissue stability concerns, providing a predictable restorative solution.

**Clinical Considerations:**

A 66‐year‐old patient presented with a failed anterior bridge and required a treatment approach that minimized surgical interventions due to pre‐existing medical conditions. Tooth 11 was extracted and replaced with an immediate implant, while tooth 21 was managed with SGET with the Benex system to achieve controlled coronal extrusion. Following stabilization, a fiber post and composite core were placed, and the tooth was prepared according to BOPT principles before final restoration with a zirconia crown. The contralateral incisor was rehabilitated with an implant‐supported crown. Clinical and radiographic evaluations at 6 months demonstrated successful preservation of the natural tooth with favorable esthetic and functional outcomes. Radiographs confirmed stable marginal bone levels, and the final photographs showed excellent soft tissue integration and patient satisfaction.

**Conclusions:**

The combination of SGET and BOPT provides a minimally invasive, cost‐effective, and time‐efficient alternative to implant therapy in cases involving significant tooth structure loss. Although long‐term clinical data remain limited, this approach shows promise in preserving natural dentition, enhancing soft tissue stability, and achieving predictable esthetic results.

## 1 Introduction

In the era of implantology, teeth with deep subgingival fractures are typically considered nonrestorable or require adjunctive treatment to establish an adequate ferrule effect before restoration [1, 2]. Although evidence supporting the necessity of a ferrule is limited, most clinicians continue to follow guidelines established during the nonadhesive cementation times [1, 3].

In order to reestablish the ferrule and to avoid violating biological width and preventing excessive subgingival preparations, various techniques have been introduced to preserve sufficient tooth structure. These include surgical crown lengthening, orthodontic extrusion, and surgical extrusion. Among these, surgical crown lengthening is the fastest approach and can be effective, but it often results in an unfavorable gingival architecture and compromised esthetics, particularly in the anterior region [4, 5, 6]. Orthodontic extrusion offers a conservative alternative, though it often involves a lengthy treatment time, higher costs, risk of relapse, and requires patient compliance [7, 8, 9]. In cases involving previously endodontically treated teeth or when orthodontic intervention is needed, surgical extrusion can be a viable option. Although not a new procedure [10, 11, 12], surgical extrusion has undergone several modifications. One major limitation of classic surgical extrusion is the need for adequate luxation, which can compromise the alveolar bone and lead to bone resorption [11, 13, 14].

To overcome these limitations, alternative techniques have been introduced [11, 13, 15]. One such approach utilizes an atraumatic extraction device, such as the Benex root extraction system (Helmut Zepf Medizintechnik GmbH), which allows for controlled vertical extrusion of the dental root, facilitating immediate post‐and‐core restoration followed by a definitive crown. The substance‐gain extrusion technique (SGET) represents a refined form of surgical extrusion aimed at optimizing restorative outcomes. Unlike conventional surgical extrusion, SGET is combined with the biologically oriented preparation technique (BOPT) to simultaneously fulfill both functional and biological treatment objectives [16, 17].

BOPT is a prosthetic preparation concept that eliminates the traditional preparation margins, allowing the restoration to guide soft tissue adaptation and support [18]. By promoting soft tissue thickening and enhancing peri‐cervical stability, BOPT improves the long‐term integrity of the periodontal interface. This approach is particularly beneficial in cases where the extruded tooth surface is narrower, potentially compromising both soft tissue thickness and width [16, 18].

This manuscript aims to describe the SGET technique through a case report in which a central incisor was restored using SGET in combination with BOPT preparation and a zirconia crown, while the contralateral incisor was treated using the conventional approach of immediate implant placement.

## 2 Material and Methods—Case Report

### 2.1 Patient Information

**FIGURE 1:**
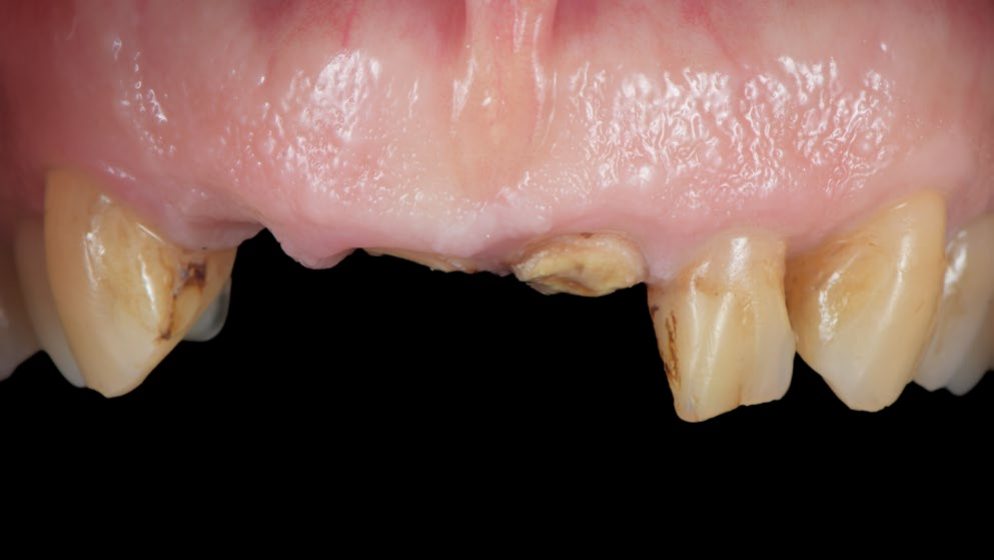
Initial clinical situation: Initial clinical situation demonstrating significant loss of tooth structure following the fracture of the anterior bridge.

A 66‐year‐old patient presented to our department (Department of Reconstructive Dentistry, University of Zürich) with a failed bridge in the anterior region (Figure 1). The restorations were approximately 15 years old, with endodontic treatment performed at the same time.

The patient, diagnosed with Post‐Polio Syndrome, expressed a preference for minimizing surgical interventions due to potential interactions between local anesthetic agents and his condition. He was a non‐smoker, had no history of periodontitis (classified as a periodontally healthy patient according to the 2018 AAP/EFP classification [19]), maintained good oral hygiene, and attended regular dental hygiene appointments.

After clinical assessment and cone beam computed tomography (CBCT) examination (3D Accuitomo 170, Morita, Japan), consultation with an endodontic specialist determined that tooth 11 was nonrestorable due to a vertical longitudinal fracture. Tooth 21 required intervention, including endodontic treatment and extrusion. For esthetic reasons, crown lengthening was not a suitable option, and the patient preferred a treatment plan that minimized duration and risk. After thorough discussion of the alternatives, we opted for a SGET for tooth 21 and extraction with immediate implant placement at position 11, following a delayed loading protocol due to the distal cantilever.

### 2.2 Intervention—Immediate Implant Placement With Concomitant Subepithelial Connective Tissue Graft

After administering local anesthesia (Ubistesin Forte, 3M ESPE, Germany), tooth 11 was carefully extracted using a minimally traumatic approach to preserve the integrity of the buccal wall. Following the extraction, the socket was thoroughly rinsed and manually cleaned. A dental implant (Primetaper Implant (Dentsply Sirona, USA), 9 mm × 4.1 mm) was placed using a surgical guide (SMOP, Swissmeda guide) in the prosthetically optimal position on the palatal aspect of the extraction socket. In accordance with the literature [20], the jumping gap, measuring over 2 mm, was filled with a xenograft of bovine bone origin (BioOss, Geistlich, Switzerland). The buccal area was prepared using a split‐thickness tunnel technique, and a subepithelial connective tissue graft was harvested from the palate. The graft was inserted into the buccal pouch and secured at the palatal aspect of the extraction site using a horizontal mattress suture with a polyamide suture (Dafilon 6/0, Braun, Germany). The palatal donor site was left to heal by secondary intention without additional suturing [21].

Postoperative instructions included the use of a 0.2% chlorhexidine mouth rinse twice daily for two weeks, adherence to a soft diet for 10–14 days, and avoidance of mechanical trauma to the surgical sites. For pain management, the patient was prescribed Brufen 400 mg (ibuprofen, Mylan Pharma GmbH, Switzerland) to be taken as needed. The sutures were removed 10 days after surgery during a follow‐up appointment.

### 2.3 Technique Description (Figure 2)

**FIGURE 2:**
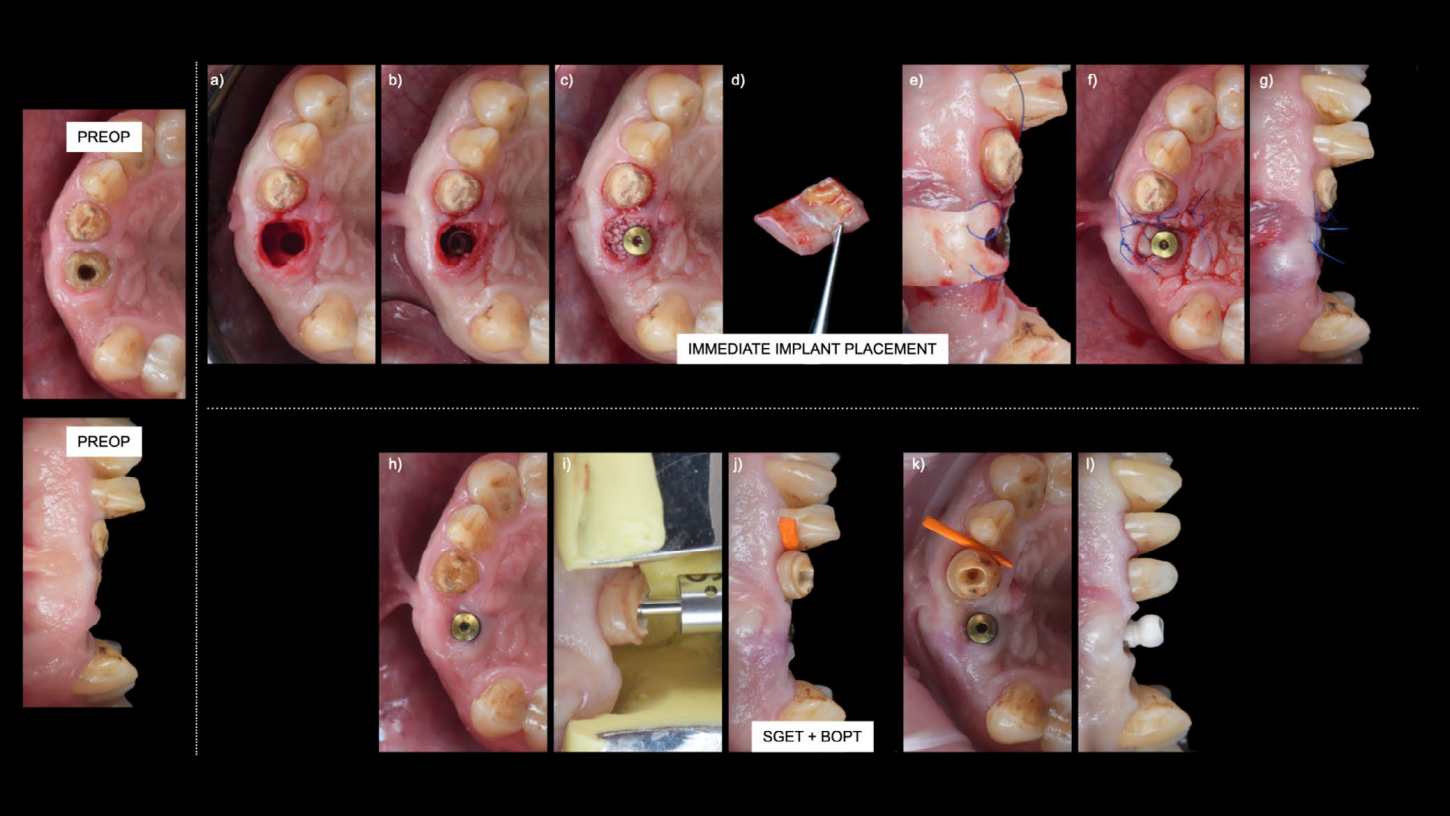
Technique workflow: (a) Osteotomy preparation for immediate implant placement, (b) immediate implant placement, (c) Augmentation of the jumping gap with xenograft, (d) harvesting of a connective tissue graft, (e) positioning of the connective tissue graft in the buccal area, (f) occlusal view of final sutures, (g) frontal view of final suturing after immediate implant placement, (h) initial clinical situation before SGET, (i) root extrusion to the desired position using the Benex system, (j, k) root stabilization with a wedge to maintain its position, (l) final impression of the implant and BOPT preparation of tooth 21. Preop—initial clinical situation, SGET + BOPT—substance‐gain extrusion technique combined with the biologically oriented preparation technique.

During the healing phase of the immediate implant, endodontic treatment was performed on tooth 21. After 3 weeks, a SGET was initiated as follows:

1. Local anesthesia (articaine 4% with 1:100,000 epinephrine—Ubistesin Forte, 3M ESPE, Germany) was administered in the region of tooth 21, and the Benex Extraction System (Helmut Zepf Medizintechnik GmbH, Seitingen‐Oberflacht, Germany) was set up according to the manufacturer's instructions.
2. An extrusion drill was positioned in the remaining root, and gentle pressure was applied using the Benex system to slowly extrude the root. (Figure 2i)
3. Once the root reached the desired level for restoration, the drill and Benex system were removed.
4. The root was stabilized using a wedge to maintain its position. (Figure 2j,k)
5. The root remnant was sealed and fixated to the adjacent tooth using flowable composite.
6. A control periapical radiograph was taken, and the patient was instructed to avoid biting on the treated area and to follow a soft diet for two weeks to minimize mechanical loading. The soft diet and avoidance of biting in the treated area were recommended to prevent micromovements of the extruded root during the initial stabilization phase. Postoperative care included the use of a 0.2% chlorhexidine mouth rinse twice daily, and analgesics (Brufen 400 mg, Mylan Pharma GmbH, Switzerland) were prescribed as needed.
7. Two weeks after the SGET procedure, a control radiograph was taken, a fiber post was cemented into the root with Panavia V5 (Kuraray Noritake Dental Inc., Japan), and a core buildup was performed using a highly filled composite.
8. The tooth was prepared using the BOPT technique, and an egg‐shell provisional restoration was placed.

After 3 months, once the emergence profile of the implant was established in the desired position and shape, tooth 21 was polished and adjacent tooth (tooth 22) was prepared for a veneer, and a final impression was taken. (Figure 2l) The final crown on the implant, including a distal cantilever, was torqued according to the manufacturer's specifications, and the crown on tooth 21 was cemented with Panavia V5 (Kuraray Noritake Dental Inc., Japan) as per the protocol. The veneer on tooth 22 was cemented using Variolink Esthetic LC (Ivoclar Vivadent, Schaan, Liechtenstein), following the manufacturer's instructions.

### 2.4 Follow‐Up and Outcomes

**FIGURE 3:**
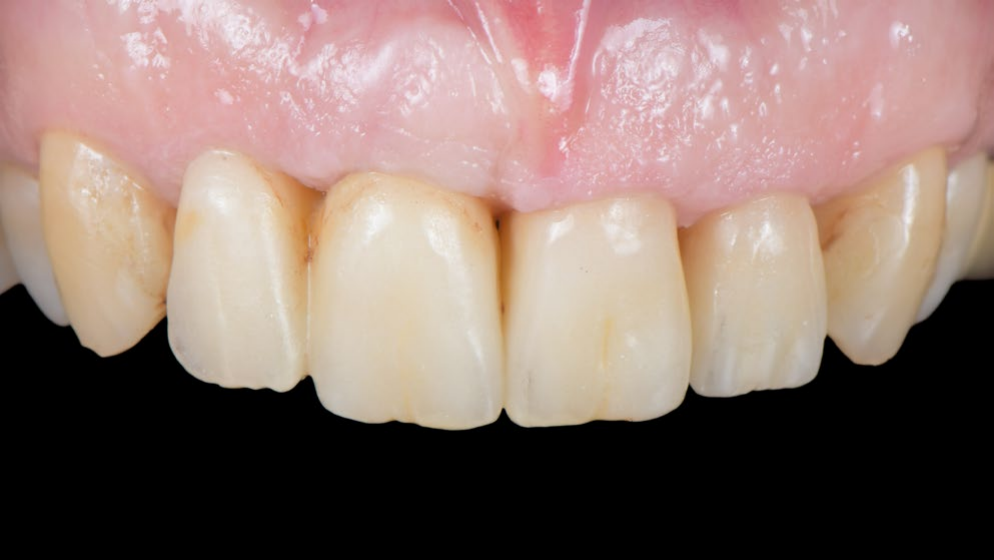
Treatment finalized: Post‐treatment outcome showing an implant‐supported restoration at position 11 with a distal cantilever, a zirconia crown on tooth 21 restored using the biologically oriented preparation technique (BOPT), and a lithium disilicate veneer on tooth 22.

**FIGURE 4:**
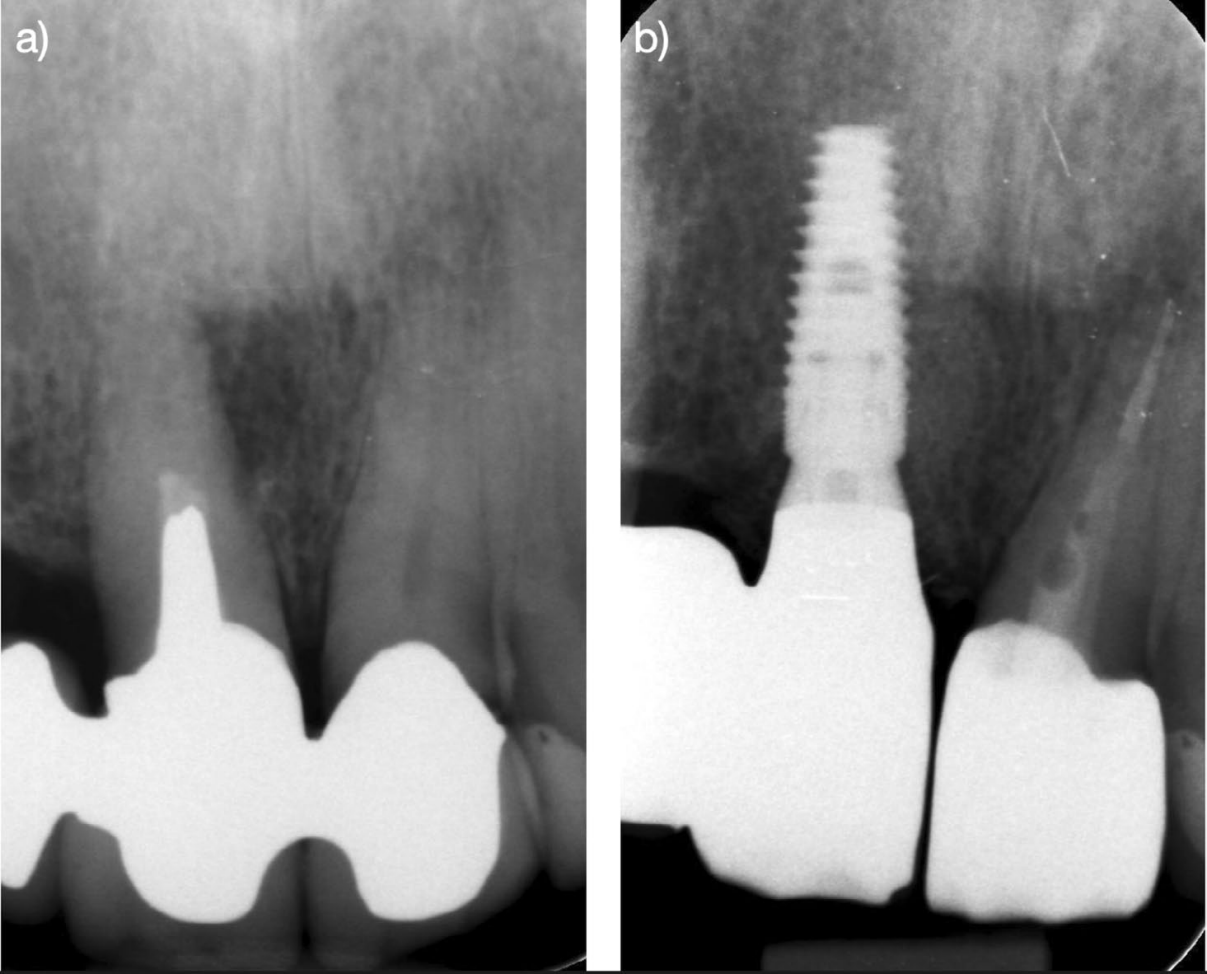
Pre‐ and post‐treatment radiographic evaluation: (a) Initial periapical radiograph showing radiolucency in the apical region of tooth 21 and insufficient endodontic treatment of tooth 11, with radiolucency present at the mesial middle portion of the root. (b) Post‐treatment periapical radiograph demonstrating radiographic bone fill around the root of tooth 21 following the substance‐gain extrusion technique (SGET), and stable marginal bone levels around the implant at position 11, supporting a distal cantilever restoration.

**FIGURE 5:**
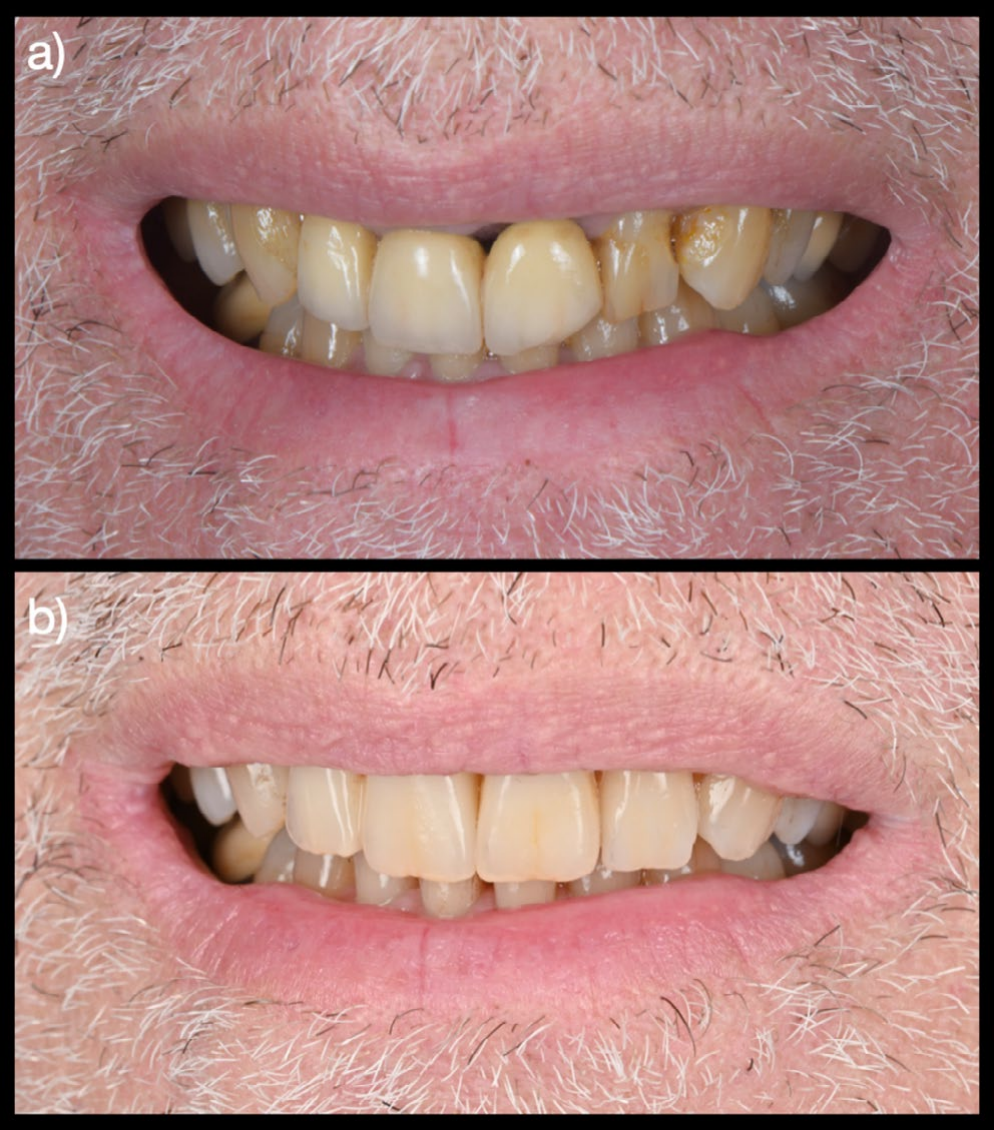
Initial and final smile photographs: (a) Initial smile photograph showing a compromised esthetic appearance and an insufficient anterior bridge; (b) final smile photograph demonstrating a harmonized and improved esthetic outcome following treatment.

The patient was followed up for 6 months, during which final photographs and radiographs were taken to establish a baseline for future assessments (Figure 3). Radiographs showed radiographic evidence of stable marginal bone levels and bone fill around the root of tooth 21 (Figure 4), although no histological analysis was performed to confirm true bone regeneration. The final photographs demonstrated an outcome that aligned with the patient's initial desires and expectations. With minimal surgical interventions, the patient's smile was not only restored but also enhanced (Figure 5). The patient expressed deep gratitude to the entire team for their efforts and the successful results.

## 3 Discussion

The SGET appears to be a straightforward and effective approach for restoring teeth with extensive loss of tooth structure. Compared to orthodontic extrusion, this technique offers a significant reduction in treatment time and cost, making it a viable alternative, particularly in cases where dental implants are not feasible [11].This is especially relevant for elderly patients with substantial tooth structure loss and pre‐existing endodontic treatments, where implant placement may not always be the optimal solution [13, 22].

One of the key advantages of SGET over orthodontic extrusion is the shorter treatment duration. Orthodontic extrusion often requires several months to achieve sufficient coronal repositioning of the tooth, followed by an additional stabilization phase [14]. In contrast, SGET allows for immediate repositioning of the tooth structure, significantly reducing the overall treatment time. This aspect aligns with the increasing demand for efficient treatment protocols, as patients today prefer minimally invasive, time‐efficient solutions that do not compromise clinical outcomes [23].

The economic aspect of treatment planning is an important factor, particularly when comparing SGET to dental implants. Patients should be fully informed about the potential costs, including long‐term maintenance expenses. According to a recent study [24], the average annual cost of implant maintenance over a 10‐year period accounts for approximately 9% of the initial treatment price [24]. This underscores the financial burden associated with implant therapy, which should be considered when evaluating alternative treatment options such as SGET.

However, due to the lack of long‐term data on the success and survival rates of SGET, direct comparisons with implant therapy remain speculative. Nevertheless, based on its restorative approach, SGET can be expected to perform similarly to conventionally surgically extruded, endodontically treated teeth restored with full‐coverage zirconia crowns. Systematic reviews report high survival rates of such restorations ranging between 90% and 95% after 5 years [25, 26].

Despite its advantages, SGET is not without potential complications. The most commonly reported risks include longitudinal root fractures and ankylosis, particularly if biomechanical principles and post‐extrusion guidelines are not carefully followed [12]. To mitigate these risks, it is crucial to adhere to established trauma and avulsion management protocols, ensuring gentle handling during extrusion and controlled stabilization of the extruded root. The authors recommend a moderate functional loading approach, where a provisional buildup is placed approximately two weeks after the SGET procedure to allow for biological adaptation and reduce the risk of mechanical overload before definitive restoration [12].

Additionally, the progressive narrowing of the root toward the apex presents esthetic and functional challenges. As the extruded portion of the tooth becomes narrower, there is an increased risk of soft tissue deficiency, unnatural crown contours, and gingival deformities such as recession. To compensate for these anatomical changes, a BOPT can be recommended [18].

The BOPT concept was introduced as a means of supporting gingival architecture and reestablishing the enamel‐cementum junction with a zirconia crown. This preparation technique eliminates the traditional finish line, allowing for improved soft tissue adaptation and increased peri‐cervical stability. Consequently, BOPT helps maintain a more natural emergence profile, addressing potential esthetic shortcomings associated with the extrusion of a narrower root structure.

Although a recent publication [27] has questioned the biological advantage of BOPT compared to conventional chamfer preparations, its clinical importance in this context extends beyond biological considerations, offering significant esthetic benefits. Given that root narrowing may lead to soft tissue instability, BOPT provides crucial support for gingival contouring and enhances the overall esthetic integration, as demonstrated in the present case.

Although long‐term clinical data on SGET is currently lacking, the technique should be considered in appropriate cases where it presents a biologically and financially viable alternative to implant therapy. Further clinical research and long‐term follow‐up studies are necessary to evaluate survival rates, complication risks, and overall clinical success.

## Disclosure

The authors have nothing to report.

## Conflicts of Interest

The authors declare no conflicts of interest.

## Data Availability

The data that support the findings of this study are available from the corresponding author upon reasonable request.
